# Relationship Between Palliative Care and Location of Death Among People With Opioid Use Disorder: A Retrospective Cohort Study

**DOI:** 10.1177/08258597251392965

**Published:** 2025-11-12

**Authors:** Lisa M. Boucher, Jenny Lau, Mary M. Scott, Karl Everett, Tara Gomes, Peter Tanuseputro, Sheila Jennings, Rebecca Bagnarol, Camilla Zimmermann, Andrew McLeod, Sarina R. Isenberg

**Affiliations:** 1 6363 Bruyère Health Research Institute, Ottawa, ON, Canada; 2 Department of Medicine, Faculty of Medicine, University of Ottawa, Ottawa,Canada; 3 Division of Palliative Care, Princess Margaret Cancer Centre, 10051 University Health Network, Toronto,Canada; 4 Division of Palliative Care, Department of Family and Community Medicine, 508783 University of Toronto, Toronto,Canada; 5 574443 The Public Health Agency of Canada, Ottawa, ON, Canada; 6 Ottawa Hospital Research Institute, Ottawa,Canada; 7 ICES, Toronto,Canada; 8 Li Ka Shing Knowledge Institute, 508783 Unity Health, Toronto,Canada; 9 Leslie Dan Faculty of Pharmacy, 508783 University of Toronto, Toronto,Canada; 10 Department of Family Medicine and Primary Care, Li Ka Shing Faculty of Medicine, 71020 University of Hong Kong, Hong Kong, China; 11 Legal Studies Department, 7984 Ontario Tech University, Oshawa, ON, Canada; 12 Disability Studies Program, 508783 Toronto Metropolitan University, Toronto, ON, Canada; 13 Division of Palliative Medicine, Department of Medicine, 508783 University of Toronto, Toronto, ON, Canada; 14 743742 Alpha House Recovery Community, Toronto, ON, Canada; 15 Renascent, Toronto, ON, Canada; 16 META:PHI, Toronto, ON, Canada

**Keywords:** palliative care, place of death, addiction—opiate, disorder—opioid related, cohort study, routinely collected health data

## Abstract

**Objective:**

People with opioid use disorder are at high risk of serious comorbidities, more likely to die from all major diseases, and their end-of-life needs remain poorly understood. We aimed to examine the relationship between receipt of palliative care across settings and the location of death among individuals with opioid use disorder.

**Methods:**

We employed a population-level cohort study using health administrative databases. Our cohort was adults with opioid use disorder in Ontario, Canada, who died between July 2015 and December 2021 (*N* = 11,200). Our exposure was receipt of palliative care within three years prior to the last 90 days of life (inpatient-only, outpatient, or none (reference)). Our primary outcome was the location of death in the community versus institution (reference).

**Results:**

Among our decedent cohort, 5636 (50.3%) received no palliative care before their last 90 days of life, and among those who received it, only 1846 (33.2%) received any outpatient palliative care and 3718 (66.8%) received inpatient-only palliative care. After covariate adjustment, receiving inpatient-only palliative care (vs no palliative care) was associated with a 6% (RR 0.94; 95% CI 0.91, 0.98) decrease in the likelihood of community death, whereas there was no relationship between outpatient palliative care and location of death.

**Conclusions:**

The association of palliative care with the location of death for people with opioid use disorder varies by setting of care. Further research is required to understand if community deaths are aligned with preferences and whether additional factors contribute to their likelihood.

## Introduction

People with opioid use disorder (OUD) are at high risk of serious comorbidities and more likely to die prematurely from all major diseases, including cancer, infectious, hepatic, cardiovascular, and pulmonary diseases.^1–3^ Due to aging populations and treatment advances for life-limiting illnesses,^4,5^ more people will experience both OUD and life-limiting illness, underscoring the need for palliative care. Yet socioeconomic disadvantages disproportionately impact people with OUD, limiting their ability to access and receive palliative care or die in preferred settings. Indeed, qualitative studies investigating the place of death in Canada and the UK show that restrictive policies and limited services interfere with desires to die at home or remain “in place” in the community for people experiencing structural vulnerability or financial hardship.^6,7^

Despite an “equity turn” within palliative care recognizing access disparities for marginalized groups,^8,9^ minimal research explores the end-of-life needs of people with OUD, including the proportion receiving palliative care and related health outcomes. Place of death is a common indicator in palliative care, with quality standards emphasizing the importance of discussing preferred settings for care and death with patients or caregivers.^10–12^ While international policies identify home as the optimal location of death, most people still die in a hospital.^7,13^ Place of death is also associated with place of care.^12,14^ In Ontario, Canada, population-level studies found that receiving palliative care, especially at home, was associated with reduced likelihood of dying in hospital.^10^^14–16^ Further, access to palliative care differs by life-limiting illness, with people who have cancer more likely to receive palliative care earlier and across multiple settings compared to people dying of organ failure or dementia.^12,17^ Palliative care research among people with OUD has focused on cancer,^18^ limiting understanding of non-cancer trajectories for this group.

Equitable access to quality palliative care requires attending to social determinants of health and specific needs of disadvantaged communities,^7^ including people with OUD. Understanding end-of-life outcomes will inform advance care planning for the location of death and better align care with patient preferences, goals, values, and needs.^19^ Thus, our study examined the relationship between palliative care receipt across settings and location of death among people with OUD. We also explored how cancer status affected this relationship, and how receiving palliative care related to other end-of-life outcomes.

## Methods

### Study Setting and Design

Ontario is Canada's most populous province with nearly 16 million residents in 2024.^20^ All registered Ontarians can access publicly funded hospital and physician services. We conducted a population-level retrospective cohort study of Ontarians who died with a recent history of OUD to evaluate the association between palliative care and end-of-life outcomes.

### Study Population

We identified Ontarians with OUD who died between July 1, 2015, and December 31, 2021. Based on health administrative database OUD case-finding algorithms,^21,22^ we identified people who met at least one of the following criteria up to 3 years before the index date (ie, 90 days before death): ≥1 emergency department (ED) visit or hospitalization associated with the International Disease Classification 10 (ICD-10) codes for OUD (F11.0–F11.9) and/or ≥1 opioid agonist treatment (OAT) prescription (see Supplementary Files: 1).^23,24^ We excluded individuals younger than 18 or older than 104 years of age, with missing or invalid sex or postal code, non-Ontario residents, those ineligible for the Ontario Health Insurance Plan (OHIP) during the 3 years prior to index, and those with no health care contact for 5 years prior to index. See our previous publication for more details about this cohort.^1^

### Data Sources

We obtained data from ICES, a non-profit research institute facilitating collection and analysis of healthcare and demographic data under Ontario's health information privacy law (see Supplementary Files: 2). The Registered Persons Database (RPDB), Ontario Marginalization Index, and Immigration, Refugees and Citizenship Canada (IRCC) Permanent Resident databases were used to identify demographic, socioeconomic, and immigration status, respectively. Acute inpatient hospitalizations, ED visits, mental health hospitalizations, and OAT prescriptions were identified using the Canadian Institute for Health Information Discharge Abstract Database (CIHI-DAD), National Ambulatory Care Reporting System (CIHI-NACRS), Ontario Mental Health Reporting System (OMHRS), and Narcotic Monitoring System (NMS), respectively. OHIP, Ontario HIV Database, and Ontario Drug Benefits (ODB) databases were used to determine decedents’ comorbidities. Cause of death was determined using Ontario Vital Statistics—Deaths Registry (ORGD), Drug and Drug/Alcohol Related Death Database (DDARD), and Ontario Cancer Registry (OCR). These datasets were linked using unique encoded identifiers and analyzed at ICES.

### Patient Characteristics

We derived demographic characteristics at index and clinical characteristics in the three years prior to index, including 13 of the most common chronic conditions (see Supplementary Files: 3).^25–39^ We used the Charlson comorbidity index as a composite measure of patient comorbidity.^40–44^ We also identified whether decedents experienced serious infections associated with injection drug use (infective endocarditis, hepatitis C prior 5 years, HIV ever),^45,46^ and benzodiazepine, stimulant, and alcohol use disorders (see Supplementary Files: 4).

### Exposure

The study exposure was receipt of ≥1 palliative care service before the last 90 days of life^47^ and after their first OUD-related event (ie, ED visit/hospitalization or OAT receipt). We categorized people according to the settings in which they received palliative care: (1) inpatient-only (including ED, inpatient units, or acute physician visits); (2) outpatient (including any physician visits in clinic, home, complex continuing care, rehabilitation, or long-term care); and (3) none (reference group) (see Supplementary Files: 5). These categories are mutually exclusive with an emphasis on receiving *any* outpatient care or *only* inpatient care; thus, individuals who received both types of care were included in the former category because it is rarer for people to receive outpatient palliative care.

### Outcomes

The primary outcome was the location of death (community vs institution). “Institution” included hospital, ED, complex continuing care, rehabilitation, or long-term care. All others were considered community deaths. As in previous work,^10^ we considered hospice deaths as community deaths, as these occur in residential-type settings and are not captured separately in the data. We considered deaths occurring on palliative care units (PCU) as institutional deaths, given that these are frequently located in hospitals in Ontario.^10^ We also examined secondary outcomes of acute care use (yes vs no)—including ED visits, hospital admissions, and intensive care unit (ICU) admissions—and intensity (total number of acute care days/visits) during the last 90 days of life.

### Statistical Analysis

To compare baseline characteristics between exposure groups, p-values were calculated using chi-squared tests for categorical variables and one-way ANOVAs or Kruskal-Wallis tests for mean (SD) and median (IQR) values, respectively, considering p-values less than 0.05 as statistically significant differences. We used modified Poisson regression models to estimate the relative risk (RR) of dying in the community and receipt of acute care, comparing those who received inpatient-only or outpatient palliative care to no receipt of palliative care. We used zero-truncated negative binomial regression to calculate incidence rate ratios (IRR) and assess the association between palliative care receipt and total acute care days. To adjust for confounding effects, we selected age, sex, rurality, income quintile, age and labour force quintile, Charlson score, and prior history of toxicity (stimulant, benzodiazepine), and alcohol-related health issues as clinically important covariates. We used interaction terms to evaluate associations between palliative care receipt and our outcomes of interest (ie, location of death, acute care use, intensity) by cancer status. We assessed multicollinearity between covariates using Variance Inflation Factors.

### Ethics Considerations

Under Section 45 of Ontario's Personal Health Information Protection Act, we did not require research ethics board approval or patient consent for this project.

## Results

During the study period, 11,200 adult decedents had a history of OUD within three years before the index. Among them, 5636 (50.3%) did not receive palliative care before their last 90 days of life. Among those who did, 1846 (33.2%) received outpatient and 3718 (66.8%) received inpatient-only care. Of people who received outpatient palliative care, 1002 (54.3%) received palliative care in clinics, 1418 (76.8%) at home, and 1367 (74.1%) also received inpatient palliative care.

### Demographics and Clinical Characteristics

Baseline demographics showed several differences across exposure groups (Table 1). Mean age of the cohort at index was highest at 62.4 (SD 16.2) for the outpatient group, 50.4 (SD 16.4) for the inpatient-only group, and 45.3 (SD 15.0) for the no palliative care group. There were sex differences across groups, with 21.2% of females receiving outpatient palliative care (vs 13.9% males), and 42.4% receiving none (vs 54.7% males). Most individuals were in the lowest two income quintiles; other indices suggest that more lived in areas with higher marginalization.

**Table 1.** Baseline (90 Days Prior to Death) Characteristics of Decedents With a History of Opioid Use Disorder (OUD) in Ontario, Categorized by the Setting of Palliative Care Received 90 Days Prior to Death.

	No. (%) of decedents				*P*-value
Characteristic	Total *N* = 11,200	Any outpatient palliative care *N* = 1846	Inpatient-only palliative care *N* = 3718	No palliative care *N* = 5636	
Sex					
Female	3995 (35.7%)	846 (21.2%)	1455 (36.4%)	1694 (42.4%)	<.0001
Male	7205 (64.3%)	1000 (13.9%)	2263 (31.4%)	3942 (54.7%)	
Age, yr					
18–44	4617 (41.2%)	252 (5.5%)	1460 (31.6%)	2905 (62.9%)	<.0001
45–54	2201 (19.7%)	275 (12.5%)	750 (34.1%)	1176 (53.4%)	
55–64	2321 (20.7%)	514 (22.1%)	814 (35.1%)	993 (42.8%)	
65–74	1108 (9.9%)	370 (33.4%)	387 (34.9%)	351 (31.7%)	
75–84	577 (5.2%)	263 (45.6%)	185 (32.1%)	129 (22.4%)	
85+	376 (3.4%)	172 (45.7%)	122 (32.4%)	82 (21.8%)	
Income quintile/Rurality					
Missing data	143 (1.3%)	14 (9.8%)	57 (39.9%)	72 (50.3%)	<.0001
Rural	1415 (12.6%)	246 (17.4%)	400 (28.3%)	769 (54.3%)	
Quintile 1 (Q1)	4233 (37.8%)	624 (14.7%)	1513 (35.7%)	2096 (49.5%)	
Q2	2120 (18.9%)	355 (16.7%)	679 (32.0%)	1086 (51.2%)	
Q3	1415 (12.6%)	257 (18.2%)	462 (32.7%)	696 (49.2%)	
Q4	988 (8.8%)	192 (19.4%)	317 (32.1%)	479 (48.5%)	
Q5 (highest)	886 (7.9%)	158 (17.8%)	290 (32.7%)	438 (49.4%)	
Age and labor force quintile					
Missing data	420 (3.8%)	33 (7.9%)	159 (37.9%)	228 (54.3%)	<.0001
Quintile 1 (Q1)	1938 (17.3%)	268 (13.8%)	638 (32.9%)	1032 (53.3%)	
Q2	2224 (19.9%)	312 (14.0%)	764 (34.4%)	1148 (51.6%)	
Q3	1888 (16.9%)	324 (17.2%)	616 (32.6%)	948 (50.2%)	
Q4	1943 (17.3%)	326 (16.8%)	630 (32.4%)	987 (50.8%)	
Q5 (highest)	2787 (24.9%)	583 (20.9%)	911 (32.7%)	1293 (46.4%)	
Material resources quintile					
Missing data	420 (3.8%)	33 (7.9%)	159 (37.9%)	228 (54.3%)	<.0001
Quintile 1 (Q1)	1122 (10.0%)	232 (20.7%)	348 (31.0%)	542 (48.3%)	
Q2	1440 (12.9%)	300 (20.8%)	471 (32.7%)	669 (46.5%)	
Q3	1610 (14.4%)	290 (18.0%)	531 (33.0%)	789 (49.0%)	
Q4	2184 (19.5%)	336 (15.4%)	666 (30.5%)	1182 (54.1%)	
Q5 (fewest resources)	4424 (39.5%)	655 (14.8%)	1543 (34.9%)	2226 (50.3%)	
Racialized and newcomer populations quintile					
Missing data	420 (3.8%)	33 (7.9%)	159 (37.9%)	228 (54.3%)	<.0001
Quintile 1 (Q1)	2149 (19.2%)	385 (17.9%)	644 (30.0%)	1120 (52.1%)	
Q2	2323 (20.7%)	410 (17.6%)	785 (33.8%)	1128 (48.6%)	
Q3	2320 (20.7%)	383 (16.5%)	717 (30.9%)	1220 (52.6%)	
Q4	2196 (19.6%)	339 (15.4%)	768 (35.0%)	1089 (49.6%)	
Q5 (highest)	1792 (16.0%)	296 (16.5%)	645 (36.0%)	851 (47.5%)	
Household dwellings quintile					
Missing data	420 (3.8%)	33 (7.9%)	159 (37.9%)	228 (54.3%)	<.0001
Quintile 1 (Q1)	753 (6.7%)	126 (16.7%)	235 (31.2%)	392 (52.1%)	
Q2	1158 (10.3%)	206 (17.8%)	368 (31.8%)	584 (50.4%)	
Q3	1545 (13.8%)	293 (19.0%)	426 (27.6%)	826 (53.5%)	
Q4	2541 (22.7%)	411 (16.2%)	830 (32.7%)	1300 (51.2%)	
Q5 (highest)	4783 (42.7%)	777 (16.2%)	1700 (35.5%)	2306 (48.2%)	
Immigrant status	371 (3.3%)	62 (16.7%)	98 (26.4%)	211 (56.9%)	0.0136

Based on the Charlson score, comorbidity severity was moderate among people receiving outpatient palliative care and mild among those not receiving palliative care (Table 2). Cancer was more common among those receiving outpatient compared to those receiving inpatient-only palliative care (48.6% vs 27.2%). A history of toxicity from other substances (benzodiazepine, stimulant, or alcohol) was more common among those receiving inpatient-only palliative care.

**Table 2.** Characteristics Related to Comorbidities and Chronic Conditions of Decedents With a History of Opioid Use Disorder (OUD) in Ontario, Categorized by the Setting of Palliative Care Received 90 Days Prior to Death.

	No. (%) of decedents				*P*-value
Characteristic	Total *N* = 11,200	Any outpatient palliative care *N* = 1846	Inpatient-only palliative care *N* = 3718	No palliative care *N* = 5636	
Charlson score for the prior 3 years					
Mean (SD)	2.21 (2.45)	3.93 (2.69)	1.93 (2.18)	1.13 (1.77)	<.0001
Median (Q1-Q3)	1 (0-3)	3 (2-6)	1 (0-3)	0 (0-2)	<.0001
Number of active comorbidities in the prior 3 years					
0	1581 (14.1%)	27 (1.7%)	181 (11.4%)	1373 (86.8%)	<.0001
1	2266 (20.2%)	155 (6.8%)	499 (22.0%)	1612 (71.1%)	
2	2491 (22.2%)	308 (12.4%)	861 (34.6%)	1322 (53.1%)	
3	1662 (14.8%)	329 (19.8%)	713 (42.9%)	620 (37.3%)	
4	1168 (10.4%)	320 (27.4%)	492 (42.1%)	356 (30.5%)	
5	822 (7.3%)	249 (30.3%)	386 (47.0%)	187 (22.7%)	
6	531 (4.7%)	186 (35.0%)	249 (46.9%)	96 (18.1%)	
7+	679 (6.1%)	272 (40.1%)	337 (49.6%)	70 (10.3%)	
Chronic conditions in the prior 3 years					
Cancer	2166 (19.3%)	1052 (48.6%)	589 (27.2%)	525 (24.2%)	<.0001
Congestive heart failure	1246 (11.1%)	428 (34.3%)	609 (48.9%)	209 (16.8%)	<.0001
Coronary artery disease	838 (7.5%)	249 (29.7%)	394 (47.0%)	195 (23.3%)	<.0001
Chronic obstructive pulmonary disease	1929 (17.2%)	572 (29.7%)	867 (44.9%)	490 (25.4%)	<.0001
Dementia	142 (1.3%)	60 (42.3%)	46 (32.4%)	36 (25.4%)	<.0001
Diabetes	2167 (19.3%)	566 (26.1%)	966 (44.6%)	635 (29.3%)	<.0001
Hepatitis C^A^	1670 (14.9%)	303 (18.1%)	861 (51.6%)	506 (30.3%)	<.0001
HIV^B^	237 (2.1%)	47 (19.8%)	104 (43.9%)	86 (36.3%)	<.0001
Infective endocarditis	354 (3.2%)	88 (24.9%)	242 (68.4%)	24 (6.8%)	<.0001
Mental health	4405 (39.3%)	783 (17.8%)	2026 (46.0%)	1596 (36.2%)	<.0001
Mood disorder	6127 (54.7%)	989 (16.1%)	2252 (36.8%)	2886 (47.1%)	<.0001
Renal disease	1830 (16.3%)	562 (30.7%)	1006 (55.0%)	262 (14.3%)	<.0001
Stroke	262 (2.3%)	101 (38.5%)	110 (42.0%)	51 (19.5%)	<.0001
Other substance use					
Benzodiazepine toxicity	216 (1.9%)	28 (13.0%)	121 (56.0%)	67 (31.0%)	<.0001
Stimulant toxicity	1085 (9.7%)	84 (7.7%)	594 (54.7%)	407 (37.5%)	<.0001
Alcohol	1988 (17.8%)	284 (14.3%)	1015 (51.1%)	689 (34.7%)	<.0001

A Chronic condition prior 5 years.

B Chronic condition ever.

Cause of death also differed across exposure groups (Table 3). Decedents receiving outpatient palliative care were more likely to die from cancer (48.2% vs 24.1% in inpatient-only or 27.7% in no palliative care groups), while those receiving inpatient-only or no palliative care had more sudden deaths, especially opioid toxicity (29.2% and 68.1%, respectively, vs 2.8% in the outpatient group).

**Table 3.** Cause of Death Types for Decedent Cohort, Categorized by Setting of Palliative Care Received 90 Days Prior to Death.

	No. (%) of decedents				*P*-value
Characteristic	Total *N* = 11,200	Any outpatient palliative care *N* = 1846	Inpatient-only palliative care *N* = 3718	No palliative care *N* = 5636	
Cause of death type					
Missing/unsure^A^	3443 (30.7%)	596 (17.3%)	1332 (38.7%)	1515 (44.0%)	<.0001
Cancer-related^B^	1682 (15.0%)	811 (48.2%)	405 (24.1%)	466 (27.7%)	
Frailty	236 (2.1%)	51 (21.6%)	105 (44.5%)	80 (33.9%)	
Other^C^	178 (1.6%)	31 (17.4%)	67 (37.6%)	80 (44.9%)	
Organ failure	1325 (11.8%)	224 (16.9%)	563 (42.5%)	538 (40.6%)	
Opioid-related sudden death	3753 (33.5%)	105 (2.8%)	1094 (29.2%)	2554 (68.1%)	
Sudden death (other)	583 (5.2%)	28 (4.8%)	152 (26.1%)	403 (69.1%)	

A There are high numbers in this category for multiple reasons, including that some individuals had no cause of death data available, including no data in ORGD or OCR, or other data discrepancies (eg, no ICD Z* code, RPDB ±1 day from ORGD); however, these reasons are the same across exposure groups.

B Cancer-related deaths were obtained from OCR and superseded other categories, with additional cancer-related deaths obtained from ORGD and re-classified into this category.

C Includes deaths from ORGD that were classified as Other, as well as any that remained as Terminal illness after re-classifying organ failure and cancer-related deaths.

### Location of Death

People with OUD receiving outpatient palliative care were nearly as likely to die in the community (46.6%) as in an institution (53.3%). Whereas those who received inpatient-only or no palliative care were more likely to die in the community (55.6% and 65.8%, respectively) versus institutions (44.4% and 34.2%, respectively; Table 4).

**Table 4.** Location of Death for Decedents With a History of Opioid Use Disorder (OUD) in Ontario, Categorized by Setting of Palliative Care Received 90 Days Prior to Death.

	No. (%) of decedents			*P*-value
Characteristic	Any outpatient palliative care *N* = 1846	Inpatient-only palliative care *N* = 3718	No palliative care *N* = 5636	
Location of death				
Community	861 (46.6%)	2067 (55.6%)	3711 (65.8%)	<.0001
Institution	985 (53.3%)	1651 (44.4%)	1925 (34.2%)	
Complex continuing care or rehabilitation	113 (6.1%)	72 (1.9%)	49 (0.9%)	
Hospital	659 (35.7%)	1472 (39.6%)	1803 (32.0%)	
Palliative care unit	78 (4.2%)	41 (1.1%)	31 (0.6%)	
Long-term care	135 (7.3%)	66 (1.8%)	42 (0.7%)	

Unadjusted and adjusted models for all outcomes are shown in Table 6. Adjusting for covariates, we found that receiving inpatient-only palliative care was associated with a 6% (RR 0.94; 95% confidence interval [CI] 0.91-0.98) decrease in the likelihood of community death compared to people who did not receive palliative care before their last 90 days of life. This relationship remained for people without cancer (RR 0.94; 95% CI 0.91-0.98), with no association among people with cancer (RR 0.97; 95% CI 0.86-1.10). No relationship existed between location of death and receipt of outpatient palliative care (RR 0.95; 95% CI 0.89-1.01), but there was an observed reduction in likelihood of community death for people without cancer (RR 0.85; 95% CI 0.79-0.93) and an observed increase in likelihood for people with cancer (RR 1.12; 95% CI 1.01-1.25).

### Acute Care Use and Intensity

With respect to other end-of-life outcomes, acute care use was more common and intense among people with OUD who received any palliative care versus no palliative care before their last 90 days of life (Table 5). However, there was no relationship between receiving outpatient palliative care and acute care use in the last 90 days of life after covariate adjustment (Table 6; RR 1.04; 95% CI 0.99-1.08). With cancer as an interaction term, those without cancer were more likely to use acute care (RR 1.11; 95% CI 1.06-1.16), while those with cancer were less likely (RR 0.90; 95% CI 0.84-0.95). People who received inpatient-only palliative care were more likely to use acute care in the last 90 days of life (RR 1.12; 95% CI 1.09-1.16); which only held for those without cancer (RR 1.14; 95% CI 1.10-1.17), with no association among those with cancer (RR 1.04; 95% CI 0.99-1.11).

**Table 5.** Palliative and Acute Care Use in the Last 90 Days of Life for Decedents With a History of Opioid Use Disorder (OUD) in Ontario, Categorized by Setting of Palliative Care Received 90 Days Prior to Death.

	No. (%) of decedents			*P*-value
Characteristic	Any outpatient palliative care *N* = 1846	Inpatient-only palliative care *N* = 3718	No palliative care *N* = 5636	
Palliative care use	1579 (85.5%)	1902 (51.2%)	1582 (28.1%)	<.0001
Acute care use				
Emergency department (ED) visit	1320 (71.5%)	2852 (76.7%)	3575 (63.4%)	<.0001
Hospital admission	1173 (63.5%)	2034 (54.7%)	1945 (34.5%)	<.0001
Intensive care unit (ICU) admission	405 (21.9%)	1219 (32.8%)	1238 (22.0%)	<.0001
Any acute care (hospital or ED)	1385 (75.0%)	2899 (78.0%)	3622 (64.3%)	<.0001
Intensity of care (mean (SD))				
Palliative care (days)	28.36 (27.89)	8.47 (12.71)	7.12 (10.73)	<.0001
ED (visits)	2.35 (2.05)	2.90 (3.57)	2.13 (2.15)	<.0001
Hospital (days) – (total inpatient Length of Stay (LOS) including ICU)	17.76 (18.11)	16.42 (17.26)	12.02 (14.45)	<.0001
ICU (days)	8.45 (9.29)	8.73 (10.13)	7.78 (9.18)	0.0462
Acute care (days) – Hospital (total LOS) + ED (1 visit = 1 day)	17.29 (18.15)	14.37 (16.85)	8.56 (12.54)	<.0001

**Table 6.** Death in the Community, Any Acute Care and Total Acute Care Days of Decedents With a History of Opioid Use Disorder (OUD) in Ontario, Categorized by Setting of Palliative Care Received 90 Days Prior to Death (Any Outpatient or Inpatient-Only vs None (Reference)).

	No. (%) of decedents							
Characteristic	Unadjusted RR^A^/IRR^B^ for all patients (95% CI)		Adjusted RR/IRR for all patients (95% CI)		Adjusted RR/IRR for patients without cancer (95% CI)		Adjusted RR/IRR for patients with cancer (95% CI)	
Death in the community (RR)								
No palliative care	1.00		1.00		1.00		1.00	
Any outpatient palliative care	0.71	(0.67; 0.75)	0.95	(0.89; 1.01)	0.85	(0.79; 0.93)	1.12	(1.01; 1.25)
Inpatient-only palliative care	0.84	(0.82; 0.87)	0.94	(0.91; 0.98)	0.94	(0.91; 0.98)	0.97	(0.86; 1.10)
Any acute care (yes or no) (RR)								
No palliative care	1.00		1.00		1.00		1.00	
Any outpatient palliative care	1.17	(1.13; 1.21)	1.04	(0.99; 1.08)	1.11	(1.06; 1.16)	0.90	(0.84; 0.95)
Inpatient-only palliative care	1.21	(1.18; 1.25)	1.12	(1.09; 1.16)	1.14	(1.10; 1.17)	1.04	(0.99; 1.11)
Total acute care days (IRR)								
No palliative care	1.00		1.00		1.00		1.00	
Any outpatient palliative care	2.37	(2.13; 2.63)	1.49	(1.45; 1.56)	1.85	(1.59; 2.14)	1.03	(0.84; 1.26)
Inpatient-only palliative care	1.9	(1.75; 2.06)	1.39	(1.36; 1.42)	1.60	(1.46; 1.76)	1.16	(0.93; 1.44)

A Relative risk.

B Incidence rate ratio.

Models adjusted for age, sex, rurality, income quintile, age and labour force quintile, Charlson score, and prior history of toxicity (stimulant, benzodiazepine), and alcohol-related health issues.

Similarly, in the adjusted model (Table 6), people who previously received outpatient palliative care had more total acute care days in the last 90 days of life (IRR 1.49; 95% CI 1.45-1.56), including for those without cancer (IRR 1.85; 95% CI 1.59-2.14) but not with cancer (IRR 1.03; 95% CI 0.84-1.26). Likewise, receiving inpatient-only palliative care was associated with more acute care days (IRR 1.39; 95% CI 1.36-1.42), but only for people without cancer (IRR 1.60; 95% CI 1.46-1.76), showing no association among those with cancer (IRR 1.16; 95% CI 0.93-1.44).

### Sensitivity Analysis

Given differences across exposure groups and implications for palliative care need and opportunity, we conducted a sensitivity analysis to remove individuals with sudden deaths (opioid toxicity or other causes) or those with missing/unsure cause of death. Among the remaining 3322 decedents in the adjusted model, our main finding did not significantly change (see Supplementary Files: 6). However, those without cancer who received any outpatient palliative care were no longer more likely to die in an institution (RR 0.88; 95% CI 0.71-1.08).

## Discussion

### Main Findings of the Study

This population-based study found a relationship between receiving palliative care and the location of death among people with OUD. After demographic and clinical covariate adjustments, compared to people not receiving palliative care before their last 90 days of life, receiving inpatient-only palliative care was associated with a 6% decrease in the likelihood of community death, holding among those without cancer. Conversely, receiving outpatient palliative care had no overall relationship to place of death, yet cancer had a modifying effect in which people with OUD and cancer were more likely to die in the community. We found similar trends for other end-of-life outcomes, with more acute care use and intensity among both palliative care groups for people without cancer. Only those with cancer who received outpatient palliative care were less likely to use end-of-life acute care.

### What This Study Adds

Receiving inpatient-only palliative care may be associated with more institutional deaths because goals of care for these individuals are more acute, consistent with our prior finding that people with OUD were more likely to receive palliative care for acute illnesses, like sepsis.^1^ Differences across groups also suggest that the likelihood of receiving palliative care increased with the number of chronic conditions. Comorbidity data indicated that the inpatient-only group had higher mental health and substance use burden, while the outpatient group had more common life-limiting conditions (particularly cancer), and the no palliative care group had the fewest comorbidities. Relatedly, almost one third of individuals receiving inpatient-only palliative care died of opioid toxicity—a sudden cause of death that limits palliative care opportunities, especially in clinic and community settings. This finding may explain why only 51.2% of this group received palliative care in their last 90 days of life, though gaps in continuity of care likely contributed. The no palliative care group had the highest opioid toxicity deaths and were most likely to die in the community, reflecting that most such deaths occur outside hospitals, and comprise younger individuals and men.^48^ Decedents with OUD tend to be younger than typical palliative care patients,^1^ as reflected in the older age of those receiving outpatient palliative care compared to inpatient-only or no-care groups. Finally, there were differences between women and men across groups, possibly reflecting greater healthcare-seeking behaviours and palliative care access among women.^47,49^ Together, these demographic and clinical differences may reflect differences in serious illness and palliative care needs. It is important to note, however, that individuals with “sudden death” causes can still be suitable candidates for palliative care, as sudden deaths can involve protracted dying periods, including hospitalization and receipt of palliative care.^50,51^ Further, premature opioid toxicity deaths occur among patients who are receiving palliative care for prior life-limiting illness.^52^

Structural vulnerability likely contributed to our findings. Primarily, people with OUD are less likely to have regular primary care compared to the general population and rely on the ED for care, including palliative care.^53,54^ Limited resources, precarious housing, and a lack of family caregivers further constrain their ability to receive care and die at home.^6^ While the place of death is a common quality metric, researchers have questioned its relevance for not directly assessing care quality.^55,56^ Moreover, among Canadians, preferences for dying at home appear more nuanced than assumed.^57^ So, the high proportion of community deaths may not reflect quality or preferences, and these settings include precarious housing (eg, emergency shelter) and public places. Although some studies suggest that materially deprived individuals prefer hospital deaths,^7,58^ people with OUD may avoid institutional settings due to concerns of inadequate pain management or stigma.^59,60^

Overall, cancer affects the relationship between palliative care and the location of death for people with OUD. Palliative care is two to three times more accessible for people with cancer across all health sectors.^12,47,61^ Palliative care is more integrated into cancer care compared to other life-limiting illness, with prognostication and illness trajectories that are more predictable, allowing earlier care initiation.^15,17,22,62^ Our findings indicate that community deaths were more common among people with OUD receiving outpatient palliative care before their last 90 days of life, yet only if they had cancer. This finding may relate to greater comorbidities and older age in this group, suggesting they were sicker and had opportunities to engage in palliative care and connect with supports outside the hospital. Most of this group continued receiving palliative care during their last 90 days of life, with more total days and less frequent acute care use. In contrast, people with OUD without cancer were more likely to die in institutions and use acute care near the end of life. These findings align with general population research showing cancer diagnoses are associated with increased access to community-based palliative care and deaths.^63,64^

### Strengths and Limitations

Health administrative data from the largest province in Canada allowed trend identification despite small numbers of decedents with OUD. While ICD-10 code Z51.5 (ie, palliative care encounters) has shown poor validity in capturing only specialty palliative care services (vs palliative intent) in past studies,^65^ we used additional sources to capture inpatient palliative care to address this limitation. We also included hospice deaths as community-based deaths and PCU deaths as institutional deaths, although definitions may vary across studies. In addition, we included long-term care in the institutional death category, yet definitions vary. Further, our OUD definition only approximates OUD and is yet to be validated in ICES data; however, it encompasses contact with the healthcare system related to OUD or its treatment, meaning that its specificity is likely high.^21^ Also, we were unable to capture non-OHIP provider visits, people without OHIP cards, and homelessness status due to the low sensitivity of the ICES algorithm and that people without fixed addresses might not be captured in census data. Moreover, people with OUD may avoid healthcare institutions due to systemic discrimination,^59,60^ and while we controlled for income and labour force participation, these are neighborhood-level rather than individual-level variables. Recognizing these limitations, we are conducting complementary qualitative research and have engaged with community members who may not be captured in the health administrative data.

## Conclusions

Only people with OUD and cancer were more likely to die in the community if they received outpatient palliative care before their last 90 days. Overall, dying in the community may not be a quality indicator for palliative care provision for all people with OUD, as these deaths may occur due to opioid toxicity and/or in the context of structural vulnerability, including within precarious housing. Future work should examine whether dying in the community aligns with patient preferences and whether additional factors modify, mediate, or confound the relationship between locations of care and death. There is also a need to explore additional end-of-life outcomes to ensure goal-concordant and quality care for people with OUD and life-limiting illness.

## Supplemental Material

sj-docx-1-pal-10.1177_08258597251392965 - Supplemental material for Relationship Between Palliative Care and Location of Death Among People With Opioid Use Disorder: A Retrospective Cohort Study Supplemental material, sj-docx-1-pal-10.1177_08258597251392965 for Relationship Between Palliative Care and Location of Death Among People With Opioid Use Disorder: A Retrospective Cohort Study by Lisa M. Boucher, Jenny Lau, Mary M. Scott, Karl Everett, Tara Gomes, Peter Tanuseputro, Sheila Jennings, Rebecca Bagnarol, Camilla Zimmermann, Andrew McLeod and Sarina R. Isenberg in Journal of Palliative Care

## Abbreviations

ED: Emergency Department
ICD-10: International Disease Classification 10
OAT: Opioid Agonist Treatment
OHIP: Ontario Health Insurance Plan
OUD: Opioid Use Disorder
PCU: Palliative Care Unit
