# Supplementary material for: Relationship Between Palliative Care and Location of Death Among People With Opioid Use Disorder: A Retrospective Cohort Study
Source: J Palliat Care. 2025 Nov 12;41(2):166–77. doi: 10.1177/08258597251392965 (PMC13013659; doi:10.1177/08258597251392965)
Supplement: sj-docx-1-pal-10.1177_08258597251392965 - Supplemental material for Relationship Between Palliative Care and Location of Death Among People With Opioid Use Disorder: A Retrospective Cohort Study [file sj-docx-1-pal-10.1177_08258597251392965.docx]

**Supplementary Files:**

1. Opioid Use Disorder (OUD) Definition
2. ICES databases descriptions and use in study
3. Chronic conditions
4. Definitions used to identify history of substance use toxicity, infective endocarditis, hepatitis C, HIV and sudden deaths in administrative databases
5. Databases and codes used to identify palliative care settings
6. Sensitivity analysis after removal of opioid toxicity deaths from the Drug and Drug/Alcohol Related Death Database (DDARD), other sudden deaths, and missing/unsure causes of death

**1. Opioid Use Disorder (OUD) Definition**

OUD definition used in study

At least 1 of the following criteria occurring within three years before the last 90 days of life

1. ≥1 OUD-related emergency department visits;
   1. NACRS: ≥1 REGDATEs for a visit that included OUD: NACRS_DXCODE1-6 “F11.0-F11.9”; (include schedule visits, transfers to/from and not seen)
2. ≥1 OUD-related hospitalization;
   1. DAD or OMHRS: ≥1 ADMDATE for a visit related to OUD _DX10CODE1-25 “F11.0-F11.9” (include suspected dx)
3. or an OAT prescription (NMS_DIN) with a three-year lookback (all DINs from ODPRN Master Drug List (15Jan21) where OMT=’Y’)

*Legend*

DAD: Discharge Abstract Database

NACRS: National Ambulatory Care Reporting System

OHIP: Ontario Health Insurance Plan

OHMRS: Ontario Mental Health Reporting System

*Reference*

Gomes T, Kolla G, McCormack D, et al. Clinical outcomes and health care costs among people entering a safer opioid supply program in Ontario. CMAJ 2022. doi:10.1503/cmaj.220892.

**2. ICES databases descriptions and use in study**

| **ICES Databases** | **Description** | **Use in study** |
| --- | --- | --- |
| Continuing Care Reporting System (CCRS) | The CCRS is a database with demographic, clinical, functional and resource utilization information on individuals receiving continuing care services in hospitals or long-term care homes in Ontario, Canada. | Place of death (outcome) |
| Discharge Abstract Database (DAD) | The DAD includes information on all hospitalizations based on a retrospective chart review including International Classification of Diseases-10 (ICD-10) diagnoses codes (up to 16 diagnoses codes for each discharge record), procedures performed during hospitalization, physician providing care, hospital administrative information, and patients’ demographic information. | Opioid related hospitalization (cohort creation), hospitalizations (outcome), and previous hospitalization diagnosis codes for chronic conditions, place of death |
| Drug and Drug/Alcohol Related Death (DDARD) | The DDARD is a database of information from chart abstraction of coronial investigations and includes details on manner of death (accidental, suicide, undetermined) and drug concentrations from postmortem toxicology. | Opioid toxicity deaths |
| Drug Identification Number (DIN) database | The DIN provides information on drugs administered in Ontario for controlled substances and for patients eligible for publicly covered benefits. | Opioids and equivalencies |
| Ontario HIV Database (HIV) | HIV is an administrative registry of individuals with diagnosed HIV (sensitivity and specificity of 96.2% and 99.6%, respectively). | Identifying proportion of HIV-positive cases |
| Homecare Database (HCD) | The HCD holds clinical, information on assessments, admission to programs and service records of care delivered at home. | Palliative care delivered at home (outcome) |
| The Immigrant, Refugees and Citizenship Canada (IRCC) | IRCC includes immigration application records for individuals who originally landed in Ontario, Canada dating back to January 1985. The main variables in this dataset include country of citizenship, level of education, mother tongue, and landing date. New immigrants who landed in Ontario and immediately moved to another province or those who moved from another province may be missed in this data. | Identified immigrants |
| Local Health Integration Network (LHIN) data | The LHIN provides health service availability and the geographic bounds of the designated LHINs in Ontario. | Geographic distribution |
| National Ambulatory Care Reporting System (NACRS) | The NACRS holds data on visits to healthcare institutions. This includes demographics, the setting visited (e.g. day surgery, emergency department, cancer care unit), and clinical data (e.g. diagnosis, treatment). | Cohort creation – Opioid-related emergency department visits, subsequent emergency department visits, including palliative care, place of death (outcomes) |
| Narcotic Monitoring System (NMS) | The NMS database stores dispensing information about all monitored drugs since 2012, submitted to the Ontario Ministry of Health by pharmacies. | Cohort creation – Opioid Agonist Treatment (OAT), opioid prescriptions, and controlled opioid equivalents |
| National Rehabilitation Reporting System (NRS) | The NRS holds data from participating adult inpatient rehabilitation facilities and programs. | Place of death (outcome) |
| Ontario Cancer Registry (OCR) | The OCR contains information about Ontario residents diagnosed with cancer, including data on those who died of cancer. | Cause of death if cancer |
| Ontario Health Insurance Plan (OHIP) Claims Database | The OHIP database holds all billing claims paid for by the Ontario Health Insurance Plan. Each record represents the delivery of a service from a particular physician to a particular patient and includes the date, the fee paid, and the number of times it was billed. | Palliative care billings (exposure) |
| Ontario Drug Benefits (ODB) | The ODB provides information on drugs administered in Ontario to patients eligible for publicly covered benefits (those <25 or >64 years of age, or eligible for disability, income or Trillium support, or living in long-term care or other care residences, or receiving professional home/community care). | Chronic conditions - multimorbidity score |
| Ontario Marginalization Index (ONMARG) | ON-Marg is a data tool that combines a wide range of demographic indicators into four distinct dimensions of marginalization, including economic, ethno-racial, age-based and social marginalization. | Descriptive statistics |
| Vital Statistics - Ontario Registry of Deaths (ORGD) | The ORGD contains information about mortality from death certificates which are completed by physicians. | Cause of death using ICD-9 and 10 codes |
| Statistics Canada’s Postal Code Conversion File Plus (PCCF+) | This is an ICES derived macro designed to link PCCF files to other census geographic identifiers and was used to create urban/rural flags, neighbourhood income quintiles, dissemination area/enumeration area, census division, and latitude/longitude. This macro is updated according to changes in census data from which it is derived. | Converts postal code from the RPDB to determine: Rurality, Income quintile, Census division, LHIN |
| Registered Persons Database (RPDB) | The RPDB holds information on each individual who has ever had an active Ontario health card number. This data was provided by the Ministry of Health and Long-Term Care (MOHLTC). The most relevant information in this dataset are demographic information, geographic information, and eligibility of OHIP coverage. | Residents’ age, sex, postal code, death information (if applicable) |

**3. Chronic conditions**

| Chronic conditions included in the algorithm based on healthcare utilization (n=13) |
| --- |
| Cancer  Congestive Heart Failure (CHF)  Chronic Obstructive Pulmonary Disease (COPD)  Coronary Artery Disease (CAD)  Dementia  Diabetes  Hepatitis C  Human Immunodeficiency Virus (HIV)  Infective Endocarditis  (Other) Mental Health Conditions  Non-psychotic Mood and Anxiety Disorders  Renal Disease  Stroke |

**4. Definitions used to identify history of substance use toxicity, infective endocarditis, hepatitis C, HIV and sudden death in administrative databases**

Other substance harms (1+ hospitalization or ED visit within three-year lookback window)

| **Substance harm** | **Data sources and codes** | **Data source details** |
| --- | --- | --- |
| Benzodiazepine poisoning | DAD, NACRS  ICD-10: T42.4 | DAD: source = inpatient, refdate = admdate, inclsuspect = F, dxtype = alldx, acute = T  NACRS: source = ed, dedup = T, inclsuspect = F, inclscheduled = F, inclfrom_typee= T,  inclto_typeip = T, inclnotseen = T, dxtype = alldx |
| Stimulant use disorder and poisoning | DAD, NACRS  ICD-10: F14.0, F15, T43.6, T40.5 | DAD (%getdadsds): source = inpatient, refdate = ddate, inclsuspect = F, dxtype = alldx, acute = T  NACRS (%getnacrs): source = ed, dedup = T, inclsuspect = F, inclscheduled = F, inclfrom_typee= T, inclto_typeip = T, inclnotseen = T, dxtype = alldx |
| Infective endocarditis^A^ | DAD, NACRS  ICD-10: I38, I39.8, I33.9, I33.0, B37.6 |  |
| Harms associated with alcohol^B^ | DAD, NACRS, OHIP  OHIP: 291 and 303  DAD, NACRS:  F10.0, F10.1, F10.2, F10.3, F10.4, F10.5, F10.6, F10.7, F10.8, F10.9, K70.0, K70.1, K70.2, K70.3, K70.4, K70.9, K29.2, X45, Y15, X65, G31.2, G62.1, G72.1, I42.6, K85.2, K86.0, E24.4, T51.0, T51.9, R78.0, Q86.0, P04.3 | OHIP: refdate=SERVDATE, FEECODE (see 291 & 303)  DAD: source = inpatient, refdate = admdate, inclsuspect = F, dxtype = alldx, acute = T  NACRS: source = ed, dedup = T, inclsuspect = F, inclscheduled = F, inclfrom_typee= T,  inclto_typeip = T, inclnotseen = T, dxtype = alldx |

Legend: ICD-10 = International Disease Classification (ICD)-10 diagnosis codes, DAD = Discharge Abstract Database, NACRS = National Ambulatory Care Reporting System, OHIP = Ontario Health Insurance Plan

A: Weir MA, Slater J, Jandoc R, Koivu S, Garg AX, Silverman M. The risk of infective endocarditis among people who inject drugs: a retrospective, population-based time series analysis. CMAJ. 2019 Jan 28;191(4):E93-9.

B: Myran, D. T., Hsu, A. T., Smith, G., & Tanuseputro, P. (2019). Rates of emergency department visits attributable to alcohol use in Ontario from 2003 to 2016: a retrospective population-level study. CMAJ. 191(29), E804-E810.

Definition for Hepatitis C

One or more of the following within a five-year lookback:

- 1. CIHI-DAD with the following diagnosis codes:

$DAD_DX10CODE 1-10: B171, B182, B19.2, B192

- 1. NACRS discharge record with the following diagnosis codes:

$NACRS_DX10CODE 1-10: B171, B182, B19.2, B192

- 1. Hepatitis C treatment drug claims from the Ontario Drug Benefits (ODB) claim database:

| **DIN** | **Drug name** | **Subclass** | **Strength** |
| --- | --- | --- | --- |
| 02370816 | BOCEPREVIR | ANTIVIRAL AGENTS | 200MG |
| 02371448 | BOCEPREVIR & PEGINTERFERON ALFA 2B & RIBAVIRIN | ANTIVIRAL AGENTS | 200MG/80MCG |
| 02371456 | BOCEPREVIR & PEGINTERFERON ALFA 2B & RIBAVIRIN | ANTIVIRAL AGENTS | 200MG/100MCG |
| 02371464 | BOCEPREVIR & PEGINTERFERON ALFA 2B & RIBAVIRIN | ANTIVIRAL AGENTS | 200MG/120MCG |
| 02371472 | BOCEPREVIR & PEGINTERFERON ALFA 2B & RIBAVIRIN | ANTIVIRAL AGENTS | 200MG/150MCG |
| 09857396 | BOCEPREVIR & PEGINTERFERON ALFA 2B & RIBAVIRIN | ANTIVIRAL AGENTS | 200MG/150MCG |
| 02444755 | DACLATASVIR HCL | ANTIVIRAL AGENTS | 60MG |
| 02444747 | DACLATASVIR HCL | ANTIVIRAL AGENTS | 30MG |
| 02436027 | DASABUVIR & OMBITASVIR & PARITAPREVIR & RITONAVIR | ANTIVIRAL AGENTS | 250MG/75MG/50MG/  12.5MG |
| 02451131 | ELBASVIR & GRAZOPREVIR | ANTIVIRAL AGENTS | 100MG/50MG |
| 02432226 | LEDIPASVIR & SOFOSBUVIR | ANTIVIRAL AGENTS | 400MG/90MG |
| 09854791 | PEGINTERFERON ALFA-2B & RIBAVIRIN | ANTIVIRAL AGENTS | 150MCG/0.5ML & 200MG |
| 02416441 | SIMEPREVIR SODIUM | ANTIVIRAL AGENTS | 150MG |
| 02418355 | SOFOSBUVIR | ANTIVIRAL AGENTS | 400MG |
| 02456370 | SOFOSBUVIR & VELPATASVIR | ANTIVIRAL AGENTS | 400MG/100MG |
| 02467542 | SOFOSBUVIR & VELPATASVIR & VOXILAPREVIR | ANTIVIRAL AGENTS | 400MG/100MG/100MG |
| 02371553 | TELAPREVIR | ANTIVIRAL AGENTS | 375MG |
| 02452294 |  | ANTIVIRAL AGENTS | 100MG |
| 02467550 | GLECAPREVIR & PIBRENTASVIR | ANTIVIRAL AGENTS | 200MG |

Definition for HIV

• 3 OHIP physician claims in 3 years with a diagnosis code of "042", "043", or "044"

• If a person has a previous hospitalization in CIHI-DAD or OHIP claim with an HIV code (ICD-9: “042”, “043”, “044”, ICD-10: “B24”, OHIP dx code: “042”, “043”, “044”) that occurs prior to the first physician claim, then that hospitalization or OHIP date is used as the incidence/diagnosis date.

Definition for sudden death

The first three characters of the ICD-10 code for underlying cause of death in ORGD database is one of the following: E89, R95-R97, S00-S99, T00-T98, V01-V99, W00-W46, W49, W59-W60, W64-W70, W73-W81, W83-W94, W99, X00-X54, X57-X99, Y00-Y36

1. **Databases and codes used to identify palliative care settings**

| ***Service*** | ***Database*** | ***Codes*** |
| --- | --- | --- |
| ***Clinic based palliative*** | *OHIP* | - *A945 + OHIP location code “Office”: Special palliative care consultation in clinic, office, home; minimum 50 min* - *K023 + OHIP location code “Office”: Palliative care support in half hour increments; may be used to add time for longer consultations following a code for A945, or for any palliative care support visit. Exclude if patient is in hospital, long-term care (LTC), complex continuing care (CCC), or rehabilitation* - *K700: Palliative care outpatient case conference* |
| ***Home based palliative care*** | | |
| *Home MD visits* | *OHIP* | - *B966: Travel premium for palliative care (billed with B998/B996)* - *B998: Home visit for palliative care between 07:00 and 24:00* - *B997: Home visit for palliative care between 24:00 and 07:00* - *A945 + OHIP location code “Home”: Special palliative care consultation in clinic, office, home; minimum 50 min* - *K023 + OHIP location code “Home”:* Palliative Care Support (>20 min.) - *A901:* House call Assessment (< 20 min.) - A900: Complex House call Assessment   Assign any physician billings from clinic based to home-based if includes attached special visit premiums for palliative care home visit: B966, B997, or B998 (e.g. A945 w/B966, K023 w/ B966). If there are multiple special visit codes billed for the same patient by the same physician on the same day, then this hierarchy will be used: 1. Special visit premium hospital inpatient codes (most important) 2. Special visit premium long-term care codes  3. Special visit premium home codes |
| *Home Care (non-MD)* | *RAI-Home Care* | - *P2S = 1 or 2: Hospice care was provided with complete or partial adherence* - *CC3f goals of care = palliative care* |
|  | *HCD* | - *SRC_admission = 95 or 54: Service recipient code (i.e., classification) of end of life on admission* - *Service_RPC = 95 or 54: Service care goal of end of life; patient provided service under end-of-life designation* - *Residence_type = 2000: Staying in hospice or palliative care unit while receiving service* - *SRC_discharge = 95 or 54: Service recipient code of end of life on discharge* |
| ***Acute care palliative care*** | | |
| *Inpatient palliative care* | *CIHI-DAD* | - *ICD-10 Code: Z51.5* - *PATSERV = 58: main patient service of “palliative care” was responsible for care* - *PRVSERV[1-8] or INSERV[1-20] = 00121: “palliative medicine” was a provider who provided service, or an intervention service code of palliative medicine was provided* |
|  | *OHIP* | - *A945 + OHIP location code “inpatient”* - *C945: Special palliative care consult, hospital (minimum 50 min)* - *C882: Family medicine palliative care, non-emergency (routine) hospital inpatient service, <20 minutes* - *C982: Specialist palliative care, non-emergency (routine) hospital inpatient service, <20minutes* - *K023 + OHIP location code ”inpatient”: Palliative care support in half hour increments, if patient was in hospital during date of claim*   Assign any A945, C945 or k023 to inpatient if includes attached special visit premium hospital inpatient visit: *C960, C961, C962, C963, C964, C990, C992, C994, C986, C996, C991, C993, C995, C999, C997* (e.g. A945 w/C960, k023 w/ C961). If there are multiple special visit codes billed for the same patient by the same physician on the same day, then this hierarchy will be used: 1. Special visit premium hospital inpatient codes (most important) 2. Special visit premium long-term care codes  3. Special visit premium home codes |
|  | *NACRS* | - *PRVSERV [1-10] = 00121: Provider service code of palliative medicine* - *CONSULTSERV1 to CONSULTSERV3 = 00121: Consult service of palliative medicine was called* |
| ***Long-term care institutes*** |  | - *W872: Family physician palliative care subsequent visit -* Nursing home or home for the aged - Palliative Care - *W972: Specialist physician palliative care subsequent visit* - *K023 + OHIP location code “LTC”* - *W882: Family physician palliative care subsequent visit (<20 min) -* Chronic care or convalescent hospital - Palliative Care - *W982:* Palliative Care Assessment (<20 min) – Specialist, Chronic care/Convalescent - *K706:* Convalescent care program case conference (per unit)   Assign any A945, C945 or K023 to LTC if includes attached LTC special visit premium travel code: W*960, W961, W962, W963, W964, W990, W992, W994, W998, W996, W991, W993, W995, W999, W997* (e.g. A945 w/W960, K023 w/ W960). If there are multiple special visit codes billed for the same patient by the same physician on the same day, then this hierarchy will be used: 1. Special visit premium hospital inpatient codes (most important) 2. Special visit premium long-term care codes  3. Special visit premium home codes |
|  | *CCRS* | - *CCRS_P1AO = 1: Received hospice care in last 14 days* |

Legend

OHIP = Ontario Health Insurance Plan

CIHI-DAD = Canadian Institute for Health Information – Discharge Abstract Database

NACRS = National Ambulatory Care Reporting System

RAI = Residential Assessment Instrument

HCD = Home Care Data

CCRS = Continuing Care Reporting System

1. **Sensitivity analysis** after removal of opioid toxicity deaths from DDARD, other sudden deaths, and missing/unsure causes of death, for death in the community of decedents with a history of opioid use disorder (OUD) in Ontario, categorized by setting of palliative care received 90 days prior to death (any outpatient or inpatient-only vs. none (reference))

| Characteristic | No. (%) of decedents | | | | | | | |
| --- | --- | --- | --- | --- | --- | --- | --- | --- |
|  | Unadjusted RR^A^ for all patients (95% CI)  N = 3,421 | | Adjusted RR for all patients (95% CI)  N = 3,322^B^ | | Adjusted RR for patients without cancer  (95% CI) | | Adjusted RR for patients with cancer (95% CI) | |
| Death in the community (RR) (Reference = Death in an institution) | | | | | | | | |
| No palliative care | 1.00 |  | 1.00 |  | 1.00 |  | 1.00 |  |
| Any outpatient palliative care | 1.06 | (0.94; 1.18) | 1.14 | (0.99; 1.30) | 0.88 | (0.71; 1.08) | 1.28 | (1.05; 1.56) |
| Inpatient-only palliative care | 0.80 | (0.70; 0.91) | 0.80 | (0.70; 0.92) | 0.79 | (0.68; 0.93) | 0.88 | (0.69; 1.12) |

^A^Relative Risk

^B^ The difference between the Ns for unadjusted and adjusted models indicates the number excluded due to missing data for any of the covariates

Models adjusted for age, sex, rurality, income quintile, age and labour force quintile, Charlson score, and prior history of toxicity (stimulant, benzodiazepine), and alcohol-related health issues.
